# Zika virus genome biology and molecular pathogenesis

**DOI:** 10.1038/emi.2016.141

**Published:** 2017-03-22

**Authors:** Anyou Wang, Stephanie Thurmond, Leonel Islas, Kingyung Hui, Rong Hai

**Affiliations:** 1 The Institute for Integrative Genome Biology, University of California at Riverside, Riverside, CA 92521, USA; 2 Department of Microbiology and Plant Pathology, University of California at Riverside, Riverside, CA 92521, USA

## Abstract

Zika virus (ZIKV) is an emerging RNA virus in the widespread *Flavivirus* genus. Recently, ZIKV has rapidly spread around the world and has been implicated in human disease, including neurological disorders, triggering public and scientific attention. Understanding how ZIKV causes disease is the highest priority, yet little is known about this virus. Here we examine the currently published data from ZIKV studies to provide the latest understanding of ZIKV genome biology and molecular pathogenesis. The ZIKV genome evolved rapidly from the *Flavivirus* genus and diverged from the members of this genus, even within the dengue virus cluster to which ZIKV belongs. Genome variations and divergences also exist among ZIKV strains/isolates. These genome divergences might account for the uniqueness of Zika disease. ZIKV infection activates not only the antiviral immune response but also the pro-inflammatory responses associated with disease symptoms. Strikingly, ZIKV activates protein complexes that are functionally associated with disease process, such as glial cell activation and proliferation (for example, Toll-like receptors), apoptosis and cell death, and inflammation. The activation of these complexes may critically contribute to Zika disease. The novel insights into ZIKV genome divergence and disease mechanisms summarized in this review will help accelerate the development of anti-ZIKV strategies.

## INTRODUCTION

Zika virus (ZIKV) is a single-stranded positive-sense RNA arbovirus belonging to the *Flavivirus* genus of the *Flaviviridae* family, members of which cause widespread morbidity worldwide.^1^ Since the recent large outbreak in 2007, ZIKV has rapidly spread throughout South America, Central America and the Caribbean, and has been implicated in neurological disorders such as microcephaly, a condition in which the fetal brain does not properly develop.^2, 3^ ZIKV has also been linked to Guillain–Barré syndrome (GBS), an autoimmune disease that causes paralysis.^4^ The severity of the diseases associated with Zika and its rapid spread have triggered an urgent need to understand this virus.

ZIKV was first isolated in 1947 from a sentinel rhesus monkey in Uganda.^5^ In 1952, it was found in humans, and it was linked to Zika disease in 1964.^6^ However, little attention was paid to this virus until 2007, when an outbreak of ‘dengue-like illness' was reported in the Yap State of Micronesia. It was estimated that >70% of Yap State residents were infected with ZIKV.^7^ Another outbreak occurred in 2013 in French Polynesia and subsequently in other Pacific Islands.^8^ Outbreaks also occurred in New Caledonia, the Cook Islands and Easter Island,^9^ and ZIKV circulation has been reported in other Pacific islands. To date, the ZIKV outbreak in Brazil is the largest ever recorded, with an estimated 165 000 suspected and confirmed cases as of August 2016.^10^

This rapid spread of ZIKV and its implication in severe disease have prompted the scientific community to develop interventions to combat Zika disease. However, the disease mechanism is not currently understood. Here we review and analyze emerging studies of ZIKV genome biology and pathogenesis to provide insight into ZIKV molecular pathogenesis to facilitate the development of Zika disease prevention strategies. However, this review does not provide a general overview of ZIKV; such overviews have recently been published.^2, 11, 12, 13, 14, 15, 16^

## ZIKV GENOME BIOLOGY

**Figure 1:**
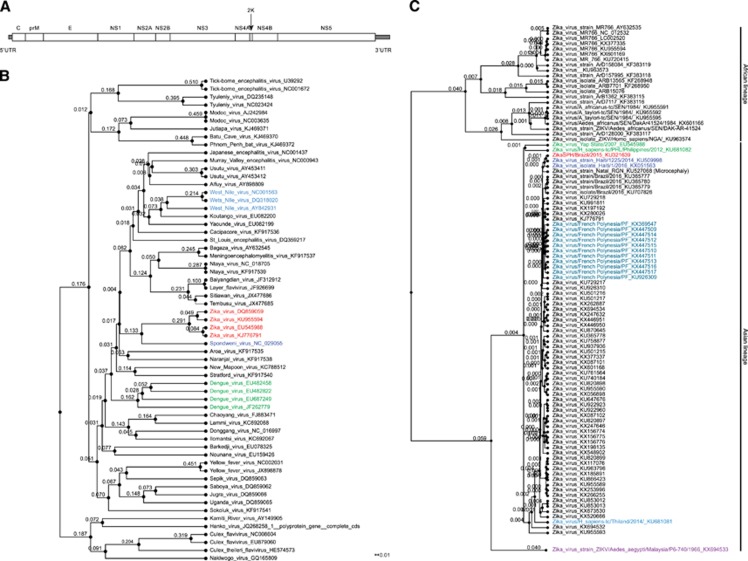
ZIKV genome divergence. (**A**) The ZIKV genome. (**B**) The ZIKV genome diverges from *Flavivirus* members. A phylogenetic tree was constructed using all gap-free sites from whole-genome sequences aligned by MAFFT (http://mafft.cbrc.jp/alignment/software/) using a bootstrap of 1000 and neighbor joining. The same method was applied to **C**. (**C**) ZIKV strain evolution based on geographic area. Zika virus, ZIKA.

The entire genome of the African prototype ZIKV strain (MR 766) was sequenced for the first time in 2007, and epidemic ZIKV strains are currently being sequenced at a rapid pace. The ZIKV genome comprises a 10.8-kb single-stranded positive-sense RNA molecule that contains an ~100 nt 5′ untranslated region (UTR), a single open reading frame of ~10 kb, and an ~420 nt 3′ UTR.^17^ The open reading frame encodes a single polyprotein, which is later processed into the capsid (C); the precursor membrane (prM); the envelope protein (E); and seven nonstructural proteins (NS1, NS2A, NS2B, NS3, NS4A, NS4B and NS5; Figure 1A). We next discuss the ZIKV genome, its divergence from other *Flavivirus* members, and the genetic variations found among various ZIKV isolates.

### Genome evolution

*Flavivirus* genus members have evolved rapidly and divergently. To understand the evolutionary relatedness of ZIKV to other members of the *Flavivirus* genus, we performed a phylogenetic analysis using the genome sequences of representative *Flavivirus* members (Figure 1B). The analysis confirmed that ZIKV clusters with dengue viruses (DENVs) at a higher hierarchical level; however, ZIKV is most closely related to Spondweni virus, resulting in an individual ZIKV cluster in the phylogenetic tree. This separation of the ZIKV cluster from other clusters at a similar hierarchical level (for example, DENV and West Nile virus (WNV)) suggested that ZIKV may have evolved disease mechanisms distinct from these other viruses despite the similarity in disease symptoms exhibited by dengue, West Nile and ZIKV-infected individuals (Figure 1B).

Another unique characteristic of ZIKV is its high homologous recombination activity. ZIKV appears to have undergone multiple recombination events between 1947 and 2007. Such active recombination is uncommon among flaviviruses and has been suggested as a mechanism for the adaptation of ZIKV to the *Aedes dalzieli* vector.^18^

Strains and isolates within the ZIKV species have also evolved rapidly. These strains can be clustered into two major lineages: African and Asian (Figure 1C). Some investigators further divide the African lineages into West African and East African lineages.^18, 19, 20^ Many of the African lineage ZIKV strains were isolated from mosquitoes (Figure 1C). The Asian lineage evolved from the Malaysia/1966 isolate. The Asian strains, which originated from the 2013 French Polynesian strain, are responsible for the recent epidemics, whereas the Brazilian isolates are most closely related to the 2014 Haitian strain (Figure 1C). The Yap State and Philippines epidemic strains originated from the same common ancestor, but the French Polynesian strain was not as closely related to the Yap isolate as originally reported (Figure 1C).^8^ Of note, a small subset of the strains included in our analysis were passaged in the laboratory prior to sequencing, and this passaging could have introduced sequence divergences. However, although passaging and other factors might contribute to divergence individually, the overall pattern presented here is likely not affected given that most of the strains are natural isolates that have not been passaged.

The genomic variation in geographically distinct ZIKV isolates accounts for the above evolutionary patterns. A total of 75 amino-acid substitutions were identified in the African lineage compared with the epidemic Asian lineage, and a total of 34 amino-acid changes and more than 400 nt variations are noted in the Asian lineage compared with the original Malaysia/1966 strain. However, the overall genome sequences are highly similar (>88.9%). The recent epidemic strains from Brazil harbor 14–18 nt variations compared with the other human strains in the same lineage. The most highly variable region in both the Asian and African lineages is the pr region of the prM protein, with ~10% amino-acid variation.^21^

In addition, genetic divergences exist in strains isolated from infected individuals. Strains isolated from fetuses with microcephaly (ZIKV2015, Natal RGN and BeH823339) carry specific mutations. ZIKV2015 harbors amino-acid mutations at polyprotein positions 550, 1143, 1259 and 2831. Natal RGN contains mutations at positions 940, 1027 and 2509, whereas BeH823339 is mutated at 7 positions (337, 354, 358, 545, 984, 1404 and 2800).^22^ However, no mutations were found that were common to all three isolates. Thus, further studies are needed to clarify the connection between these mutations and microcephaly.

Genetic differences were also found in the UTRs of the epidemic strains compared with the pre-epidemic prototype strain. Specifically, a large 9-nt bulge (UAG UCA GCC) replaces the 3′ UTR stem-loop region in epidemic ZIKV.^23^ Variations in UTRs may have an impact on viral genome stability, replicative efficiency and, consequently, overall viral fitness and transmissibility. Further experimentation is required to determine whether differential functions are associated with genome sequence variations.

Phylogenetic studies have exposed patterns of ZIKV genome evolution and variations, but these patterns are based on limited ZIKV isolate availability. With more strain sequences becoming available, the origins of ZIKV and its evolutionary pattern should become clearer. When combined with other factors, such as geography and clinical data, the global ZIKV migration pattern and ZIKV genome evolution will be elucidated.

### Functional genomics

The genomes of all members in the *Flavivirus* genus share certain common properties, and the functional genomics of ZIKV has mostly been inferred from previous work with other flaviviruses. These viruses encode the same proteins in the same order flanked by a 5′ and 3′ UTR. The 5′ UTR contains the vRNA promoter and terminates with a type I cap, followed by the conserved dinucleotide AG. The 3′ end of the transcript is not polyadenylated and is instead terminated with a conserved CU_OH._^24^

The C protein comprises the viral capsid, which is icosahedral in shape and surrounded by a spherical lipid bilayer membrane derived from the host. The M and E proteins are displayed on the viral surface and have transmembrane helices that anchor them in the outer membrane. As mature viral particles egress, the prM protein is cleaved by a furin-like protease located in the *trans*-Golgi network into the pr peptide and the M protein. It has been hypothesized that prior to this step, the prM protein participates in E protein folding.^25^

The E protein is the major virion surface protein and is involved in host cell binding and membrane fusion. The structure of the ZIKV E protein in complex with the flavivirus broadly neutralizing antibody 2A10G6 was also solved, and this antibody was shown to neutralize ZIKV infection *in vitro* and protect mice *in vivo.*^26^

The structure of the E protein exposed a potentially important difference between ZIKV and other flaviviruses. DENV is glycosylated at Asn67 on the E protein, which is important for binding to the host receptor DC-SIGN.^27^ ZIKV apparently lacks this glycosylation site but is glycosylated at Asn154 in many of the strains that have been analyzed. The DENV E protein is also glycosylated at the same location (Asn153), and this modification is beneficial for virion release.^28^ Similarly, the glycosylation of the WNV E protein enhances the virus' neurotropic virulence.^29^ The region surrounding this glycosylated area tends to vary among flaviviruses and may be an important determinant for antibody specificity.

The nonstructural proteins (NS1-NS5) form the replicative complex and play a role in host innate immunity antagonism. The ZIKV NS1 protein is highly similar to other flaviviruses, with a noted divergent electrostatic potential on the loop surface. This divergent region is involved in binding host factors and protective antibodies, so it could be a potential antiviral target. The NS1 protein has also been implicated in immune evasion and appears to play a role in viral replication along with NS4A.^30^ NS2A, NS2B, NS4A and NS4B are hydrophobic proteins that may be membrane-associated. However, they do not have any known enzymatic motifs, and their specific functions have yet to be elucidated.^23^ The NS3 protein is central to viral replication and polyprotein processing due to its N-terminal protease domain along with its C-terminal RNA helicase activity. The NS5 protein has two known activities: an RNA-dependent RNA polymerase activity performed by the C terminus and an RNA capping function executed by the methyltransferase domain located at the N terminus.^18^ NS5 suppresses IFN signaling via proteasome-dependent degradation of human STAT2.^31^

## MOLECULAR PATHOGENESIS

### Transmission

Similar to many other flaviviruses, ZIKV is mainly transmitted by a mosquito bite, and the skin is the first line of defense against infection. To date, Zika vRNA has been isolated from seven different *Aedes* species. Unlike many flaviviruses, however, there is strong evidence to indicate that ZIKV can be transmitted from human to human through many different routes, including sexual contact, blood transfusion and vertically from mother to fetus.^32^ Indeed, Zika vRNA persists in semen longer than in blood, saliva or urine. In one case study, Zika vRNA was detected in a patient's semen sample for up to 6 months after the onset of illness.^33^ Similarly, vRNA has been detected in the female genital tract, and a suspected case of female-to-male transmission has been reported.^34^ Although ZIKV RNA persists in urine and saliva, it is not yet known whether these fluids can facilitate transmission.

### ZIKV-associated diseases

Infection with ZIKV is often asymptomatic but can cause mild symptoms similar to those of dengue fever, except the breaking bone pain. During the 2013 French Polynesia outbreak, an increase in reported GBS was reported.^35^ This report marked the first time Zika was associated with severe neurological complications. There have also been reports of other Zika-associated neurological diseases.^36, 37^

There have also been more children with microcephaly and other congenital abnormalities born to ZIKV-infected women in Northeast Brazil.^38, 39^ During the French Polynesia outbreak, there were more cases of central nervous system malformations concurrent with the ZIKV epidemic.^35^ The Centers for Disease Control and Prevention therefore declared that prenatal Zika virus infection causes microcephaly; however, definitive causality remains to be established.^40, 41^

The increase in microcephaly cases in Brazil temporally associated with the recent ZIKV epidemic has driven researchers to establish causality between ZIKV and congenital neurological complications. Zika vRNA has been detected in fetal brain tissues.^42^ A ZIKV strain of African lineage, MR766, preferentially infected neural progenitor cells (NPCs) in 14-day-old forebrain organoids and caused a reduction in organoid size. In 28-day-old organoids, which contain progenitor cells in addition to NPCs, most ZIKV-infected cells were NPCs, indicating specific tropism. These brains exhibited reduced ventricular zones and neuronal layer thicknesses, resembling microcephaly.^43^

### Disease mechanisms

Consistent with mosquito bite transmission, ZIKV infects human dermal fibroblasts, epidermal keratinocytes and immature dendritic cells.^44^ The congenital abnormalities associated with ZIKV suggest that it may also be capable of bypassing the placental barrier, and *in vitro* evidence demonstrates that ZIKV is able to infect human placental macrophages and cytotrophoblasts.^45^ Animal model experiments have also demonstrated ZIKV infection of the placenta and fetal brain, resulting in diminished brain development.^42, 46, 47, 48^ No other flavivirus has definitively been shown to cause birth defects in humans, suggesting that this tropism may be specific to ZIKV. Consistent with ZIKV causing neurological disorders, ZIKV can also infect human NPCs, neurospheres and forebrain organoids.^43, 49, 50, 51^

**Figure 2:**
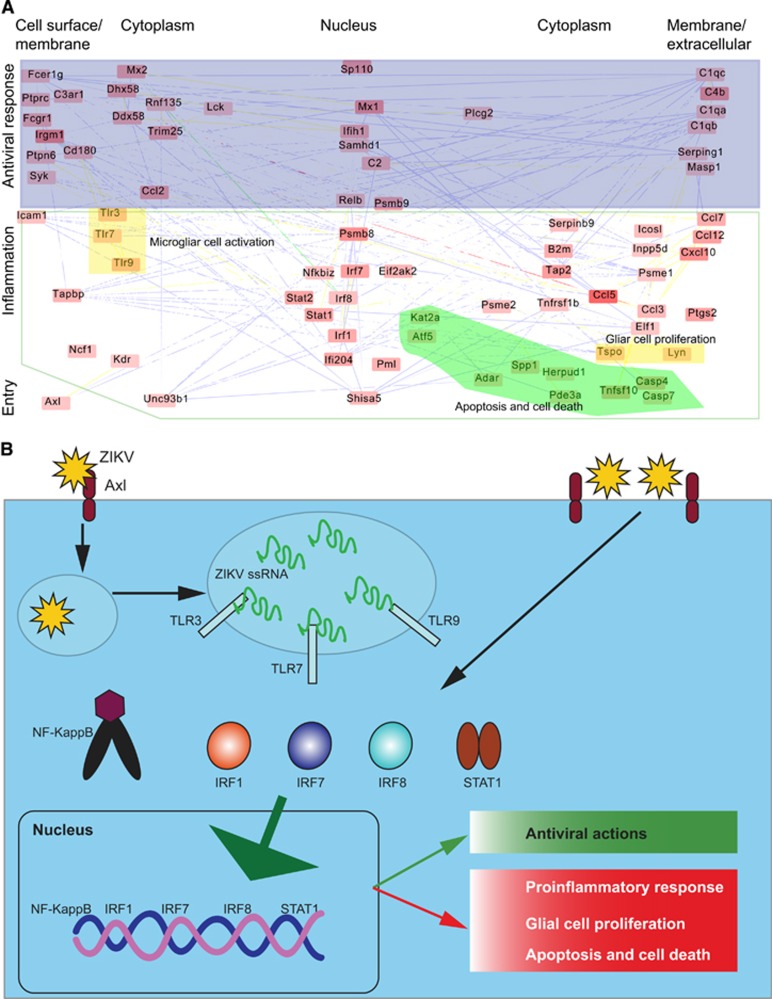
Immune response to ZIKV. (**A**) An immune response network was constructed using published RNA-seq data. The network was inferred from the systems network database as we previously described,^52^ including domains, co-localization, physical interaction and co-expression. Briefly, the protein–protein interactions were extracted from a database and subsequently enriched for significant genes derived from RNA-seq data, as described in our publication.^52^ The color of a node (gene) denotes the expression level; expression increases from light red to dark red when compared with mock controls. A line (linkage) represents the interaction type, yellow—common protein domain, blue—co-localization, green—physical interaction. For illustration, the other interactions are labeled as white as background. (**B**) A proposed scheme of the inflammation initiated by ZIKV infection leading to an antiviral response and an inflammatory response associated with diseases. Zika virus, ZIKV.

To understand the mechanism of Zika disease, we constructed a systems network based on the currently available data^47, 50^ (Figure 2A). Genes (nodes) in this network are activated by ZIKV infection and interact with each other to generate a host immune response. For illustrative proposes, we regrouped this entire network into three subnetworks: virus entry, antiviral response and pro-inflammatory complexes associated with disease development (Figures 2A and 2B).

ZIKV penetrates host cells via receptor-mediated endocytosis. Current evidence suggests that the cell-surface phosphatidylserine receptor Axl functions as the primary receptor for ZIKV entry.^44, 48^ The viral E proteins, which are arranged as dimers on the viral surface, mediate attachment to the host cell. Viral entry and replication trigger the host immune response (Figures 2A and 2B).

These immune responses could also function as antiviral and pro-inflammatory responses. The antiviral response primarily includes three modules (protein complexes): cell surface receptors (Fcer1g, Ptprc, Ptpn6, Syk and C3ar1); the innate immune response (Fcgr1, Irgm1, Ptpn6, Cd180, Sp110, Ddx58, Mx2, Mx1, Samhd1, Ifih1, Rnf135, Dhx58 and Trim25); and plasma proteins associated with the acute inflammatory response (C4b, C1qb, C1qa, Serping1, Masp1,C2 and C1qc; Figure 2A). These antiviral immune responses may also cause inflammation, leading to disease symptoms.

ZIKV also activates inflammatory modules (Figure 2A), including factors involved in the inflammatory response (Ccl12, Nfkbiz, Tnfrsf1b, Cxcl10, Ccl7, Ccl5, Ccl3 and Ccl2), the adaptive immune response (Serpinb9, Icam1 and Relb), B-cell-mediated immunity (Inpp5d, Irf7 and Icosl), the regulation of T-cell-mediated immunity (B2m and Tap2), antigen presentation (Tapbpl, Psmb8, Psmb9, Psme2 and Psme1), leukocyte-mediated immunity (Ncf1) and the regulation of cytokine production (Irf8, Irf1, Ptgs2, Elf1 and Tspo). Activating these inflammatory response modules could attract T cells and other leukocytes to the site of infection. In addition, they may enhance cytokine and chemokine production. Ultimately, the accumulation of these immune factors leads to tissue damage. Consistent with the strong inflammatory response, it has been hypothesized that ZIKV infection can cause autoimmune diseases, such as GBS.^35^

Interestingly, ZIKV also activates modules associated with apoptosis and glial cell regulation (Figure 2A); these modules include microglial cell activation (Tlr3, Tlr7 and Tlr9), regulation of glial cell proliferation (Lyn and Tspo), positive regulation of apoptosis (Stat1, Eif2ak2, Ifi204, Shisa5, Pml, Casp7, Casp4 and Tnfsf10), and regulation of programmed cell death (Kdr, Adar, Herpud1, Spp1, Kat2a, Pde3a and Atf5). The activation of these modules is consistent with reports demonstrating that ZIKV infection induces cell apoptosis and death, and dysregulates cell cycle progression.^44, 47, 49, 50, 51^

To summarize the network modules and pathways potentially involved in the diseases initiated by ZIKV, we proposed a scheme to model ZIKV molecular pathogenesis (Figure 2B). ZIKV entry and replication together activate signaling pathways such as Toll-like receptor pathways, which subsequently activate transcription factors. These transcription factors cause dysregulation of host cell transcription, leading to antiviral and pro-inflammatory responses, apoptosis and dysregulation of glial cell proliferation. This proposed model may help to elucidate the molecular mechanism behind ZIKV-induced microcephaly, in which pro-inflammatory and apoptotic pathways in pregnant mice are activated, and the size of the developing forebrain is markedly reduced.^43^

These network modules were computationally inferred from a systems network database as previously described^52^ and should be validated by further experimental data. However, many of these module components have been observed in recent reports, and most of them, such as apoptosis,^53^ are shared by other flavivirus members, but some are unique to ZIKV, such as the cell-surface receptor Axl.^44^

Although the network pattern helps us to understand the disease mechanism of ZIKV, it is limited by a small available data set and resource bias given that a considerable amount of the data were generated using immunodeficient mice. This network should be built upon and further validated by including new emerging data.

### Protection against ZIKV

Larocca *et al.* developed two conventional vaccine platforms, a DNA vaccine and a purified inactivated virus vaccine. Both vaccines were tested for efficacy in mice. Six different DNA vaccine candidates were designed, each with different frameshift mutations. The prM-Env-expressing plasmid, which was derived from the Brazil P ZIKV BeH815744 strain, elicited higher Env-specific antibody titers than the other DNA candidates and provided the most protection against homologous ZIKV infection. The immunologic mechanism of protection against ZIKV challenge was due to Env-specific IgG and was T-cell-independent. The inactivated virus vaccine also provided complete protection against viral challenge in the mouse model. However, the protective efficacy of this platform varied with the mouse strain, suggesting host-specific responses to viral infection. This varied host response warrants further investigation in human hosts, but extension of these vaccine platforms to human application is promising.^54^

ZIKV-specific antibodies are another potential therapeutic means to combat ZIKV infection. However, special care should be taken when evaluating such antibodies to avoid antibody-dependent enhancement (ADE) in individuals previously infected with DENV. ADE is a phenomenon in which non-neutralizing antibodies facilitate heterologous virus entry into host cells, and it was recently demonstrated that DENV antibodies can drive ADE of ZIKV infection.^55^ One study found that antibodies against the ZIKV E protein domain I/II (EDI/II) are cross-reactive to DENV and are poorly neutralizing. These antibodies also enhance both ZIKV and DENV infection. By contrast, EDIII-specific antibodies are potent ZIKV-specific neutralizing antibodies, and one EDIII-specific antibody protected mice from lethal ZIKV infection.^56^ This study serves as a proof of principle for antibody-based therapy and provides a template for the development of potent neutralizing ZIKV antibodies.

## CHALLENGES AND FUTURE DIRECTIONS

ZIKV has rapidly spread around the world. However, given that Zika disease symptoms resemble those of other arboviral diseases and that ZIKV infection is often mild or asymptomatic, its prevalence is likely underestimated. However, overestimation of ZIKV prevalence can also occur. Serological and molecular tests frequently produce false-positive results due to cross-reactivity and sequence similarities with other flaviviruses.^57^ Because of these complications, future studies should combine conventional clinical data with mathematical modeling and computational biology concepts to dissect the complexity of Zika disease.

In the past, *Flavivirus* disease models have relied on immunodeficient mice because many flaviviruses are not able to efficiently antagonize the mouse innate immune response, preventing viral replication. ZIKV researchers have encountered a similar problem. In addition, Zika disease severity varies with the genetic background of each mouse strain. This variation leads to the challenge of generating consistent results from ZIKV-infected mouse models. This problem is compounded when applying discoveries from mouse models to humans due to non-trivial differences in mouse and human biology. Future studies should address these differences and work to develop an authentic mechanism of ZIKV mammalian pathogenesis.

## CONCLUSION

The rapid evolution of the ZIKV genome accounts for the recent ZIKV pandemic and the uniqueness of Zika disease. The immune network is activated by ZIKV and functions in glial cell activation and proliferation, apoptosis and cell death. The promotion of inflammation critically contributes to Zika diseases. The novel molecular insights of ZIKV pathogenesis provided here will help to combat Zika diseases and eventually eliminate ZIKV.
